# Transcriptome Analysis Reveals Candidate Genes Involved in Gibberellin-Induced Fruit Setting in Triploid Loquat (*Eriobotrya japonica*)

**DOI:** 10.3389/fpls.2016.01924

**Published:** 2016-12-21

**Authors:** Shuang Jiang, Jun Luo, Fanjie Xu, Xueying Zhang

**Affiliations:** ^1^Forestry and Pomology Research Institute, Shanghai Academy of Agriculture SciencesShanghai, China; ^2^Shanghai Key Laboratory of Protected Horticultural Technology, Shanghai Academy of Agriculture SciencesShanghai, China

**Keywords:** triploid loquat, exogenous GA3, parthenocarpy, fruit setting, transcriptome analysis

## Abstract

The triploid loquat (*Eriobotrya japonica*) is a new germplasm with a high edible fruit rate. Under natural conditions, the triploid loquat has a low fruit setting ratio (not more than 10 fruits in a tree), reflecting fertilization failure. To unravel the molecular mechanism of gibberellin (GA) treatment to induce parthenocarpy in triploid loquats, a transcriptome analysis of fruit setting induced by GA_3_ was analyzed using RNA-seq at four different stages during the development of young fruit. Approximately 344 million high quality reads in seven libraries were *de novo* assembled, yielding 153,900 unique transcripts with more than 79.9% functionally annotated transcripts. A total of 2,220, 2,974, and 1,614 differentially expressed genes (DEGs) were observed at 3, 7, and 14 days after GA treatment, respectively. The weighted gene co-expression network and Venn diagram analysis of DEGs revealed that sixteen candidate genes may play critical roles in the fruit setting after GA treatment. Five genes were related to auxin, in which one auxin synthesis gene of *yucca* was upregulated, suggesting that auxin may act as a signal for fruit setting. Furthermore, ABA 8′-hydroxylase was upregulated, while ethylene-forming enzyme was downregulated, suggesting that multiple hormones may be involved in GA signaling. Four transcription factors, *NAC7*, *NAC23*, *bHLH35*, and *HD16*, were potentially negatively regulated in fruit setting, and two cell division-related genes, *arr9* and *CYCA3*, were upregulated. In addition, the expression of the GA receptor *gid1* was downregulated by GA treatment, suggesting that the negative feedback mechanism in GA signaling may be regulated by *gid1*. Altogether, the results of the present study provide information from a comprehensive gene expression analysis and insight into the molecular mechanism underlying fruit setting under GA treatment in *E. japonica*.

## Introduction

Fertilization is an important step in the development of all sexually reproducing higher plants. After pollination and successful fertilization, the static flower ovary is shifted to young fruit (Gillaspy et al., 1993). In this process, the maternal structures of the ovary become enlarged with the development of the embryo, thereby inducing fruit setting. However, in many plants, the development of fruits could be without fertilization, referred to as parthenocarpy (Gustafson, 1939), and the fruit is therefore seedless. The parthenocarpy could be natural or induced by the exogenous application of plant hormones, such as gibberellins (GAs), used in citrus, apple, and pear (Davison, 1960; Niu et al., 2015; Mesejo et al., 2016); auxins, used in watermelon (Newbury et al., 1978), and cytokinins, used in tomato and kiwifruit (David et al., 1996; Ding et al., 2013). More than a hundred GAs have been identified from plants (MacMillan, 2001). Several studies have reported that three GAs (GA3, GA4, and/or GA7) could be applied (Yarushnykov and Blanke, 2005) for the induction of parthenocarpic fruit development in Rosaceae species, such as apple (Watanabe et al., 2008), loquat (Goubran and El-Zeftawi, 1986), and peach (Kiyokawa and Nakagawa, 1972).

Exogenous GA could affect plant growth and regulation. Two genes are well known to regulate GA signaling. The GA receptor GIBBERELLIN INSENSITIVE DWARF1 (GID1) was identified to directly bind GA (Ueguchi-Tanaka et al., 2005). The other gene, DELLA, plays an important role in the negative control of GA signaling (Peng et al., 1999). In tomato, antisense silencing of the DELLA gene yielded slender-like plants with elongated flower trusses and was sufficient to overcome the growth arrest typically imposed on the ovary at anthesis, resulting in parthenocarpic fruits (Marti et al., 2007). A yeast two-hybrid analysis showed that the combination of GID1 and DELLA was possible, suggesting that the triple complex comprising GID1-GA-DELLA regulated GA signaling (Sun, 2011). Other genes have been implicated in parthenocarpy, and these genes might be the downstream genes in GA signaling. Fruit initiation in *Arabidopsis* is generally repressed until fertilization. However, mutations in AUXIN RESPONSE FACTOR8 (ARF8) uncouple fruit initiation fertilization, resulting in the formation of seedless, parthenocarpic fruit (Goetz et al., 2007). In tomato, AUXIN RESPONSE FACTOR 7 (ARF7) was identified as a modifier of both auxin and gibberellin responses during tomato fruit setting and development (De Jong et al., 2011).

Loquat (*Eriobotrya japonica* Lindl.), a member of the Rosaceae species, is an important sub-tropical fruit tree in Asia (Janick, 2013). Most loquats are diploid and have 3–5 seeds. The triploid loquat is an available germplasm with a high edible fruit rate but a very low fruit setting ratio (Liang et al., 2011a), reflecting the lack of seed in nature, and it has thicker leaves (Wang et al., 2011b), larger flowers (Liang et al., 2011b), and different pollen morphology (Guo et al., 2011) compared to diploid loquat. GA_3_ was used to spray triploid loquat fruit during peak flowering induced fruit setting, which could be commercially applicable to increase the volume of production. The key genes in loquat fruit setting with GA treatment remain unclear. In recent years, RNA-sequencing (RNA-Seq) technology has become a powerful tool for species lacking reference genome information. RNA-Seq enables rapid gene discovery and more sensitive and accurate profiles of the transcriptome than microarray analysis or other techniques. To better understand the molecular mechanisms of fruit setting with GA treatment in loquat, we used RNA-Seq technology to identify and characterize the expression of a large number of genes, particularly those differentially expressed after spraying with GA.

In the present study, we sprayed 500 mg/L of GA_3_ on the full-bloom stage of the triploid loquat, collected samples of flowers/fruits at 2 weeks after GA treatment, and investigated the transcriptome in fruit setting to reveal the molecular mechanism of GA signaling. We sequenced seven cDNA libraries using Illumina deep-sequencing technology. The assembled and annotated transcriptome sequences and gene expression profiles will provide valuable resources for the identification of loquat genes involved in fruit setting and development.

## Materials and Methods

### Plant Materials

The flowers of ‘Danhe’ triploid loquat (*E. japonica*) in the blossom period were treated on Nov. 3, 2015 at the experimental farm of Shanghai Academy of Agricultural Sciences in Zhuanhang Town (Shanghai, China). The loquat trees were 13 years old and considered to be in the adult phase. All samples were collected from the same trees at each stage. The loquat flowers at full-bloom stage were sprayed with 500 mg/L of GA_3_ for treatment (Goubran and El-Zeftawi, 1986) and double-distilled H_2_O for control. The concentration of GA_3_ was based on our preparatory experiment. We sampled at day 0 (GA untreated, P0), day 3 (CK3 and T3), day 7 (CK7 and T7), and day 14 (CK14 and T14). At each sampling point, twenty flowers/fruits were collected and used for RNA extraction. Each library contained pooled samples of equal quantities of RNA from three biological replications for each stage. A total of seven samples were used for RNA-seq.

### RNA Extraction and RNA-Seq

Total RNA was extracted using a modified CTAB method according Qian et al. (2014). RNA purity was assessed using the NanoDrop^®^ 2000 (Thermo, CA, USA). RNA concentration and integrity were assessed using the RNA Nano 6000 Assay Kit of the Agilent Bioanalyzer 2100 System (Agilent Technologies, Santa Clara, CA, USA). Genomic DNA was digested with DNase I. The cDNA libraries were constructed with approximately 5 μg of RNA for each sample using the NEBNext Ultra RNA Library Prep Kit (NEB Inc., San Francisco, CA, USA) according to the manufacturer’s instructions, and index codes were added to attribute sequences to each sample. To select cDNA fragments of preferentially 250–300 bp in length, the library fragments were purified using the AMPure XP System (Beckman Coulter, Beverly, MA, USA). Subsequently, 3 μl of USER Enzyme (NEB, USA) was used with size-selected, adaptor-ligated cDNA at 37°C for 15 min. Then PCR was performed using Q5 Hot Start HiFi DNA polymerase, Universal PCR primers and Index (X) Primer. The PCR products were purified (AMPure XP system), and library quality was assessed on the Agilent Bioanalyzer 2100 system. Each library (∼10 ng) was used for Paired-End sequencing using Illumina HiSeq^TM^ 4000 (San Diego, CA, USA). Raw sequence data in FASTQ format were filtered to remove reads containing adaptors, reads with more than 5% unknown nucleotides, and low-quality reads with more than 20% bases of quality value ≤ 10. Only clean reads were used in the following analysis. The clean reads data have been deposited in the NCBI Sequence Read Archive^1^, and the accession numbers are SRR4195876 (P0), SRR4195877 (CK3), SRR4195878 (CK7), SRR4195879 (CK14), SRR4195880 (T3), SRR4195881 (T7), and SRR4195882 (T14).

### Transcriptome Assembly and Annotations

Transcriptome *de novo* assembly was performed using the short-read assembly program Trinity. Subsequently, the redundancy of unigenes was removed using TGICL (v.2.1; Pertea et al., 2003). Based on gene family clustering, the unigenes were divided into two classes: clusters and singletons. The former was prefixed with ‘CL’ and the latter with ‘unigene.’ The transcriptome reference data (99.7 MB) was deposited in Baidu Cloud (Baidu, Beijing, China)^2^. All unigenes were annotated by searching against six public databases, including NR, NT, Swiss-Prot, Kyoto Encyclopedia of Genes and Genomes (KEGG), COG, and Gene Ontology (GO) database, using BLASTx, and the best-aligning results were used to determine the sequence direction of the unigenes. GO annotation was performed using Blast2GO software (Conesa and Gotz, 2008), and KEGG pathway annotation was performed using Blastall software against the KEGG database.

### Identification of Significantly Different Expressed Genes (DEGs)

Clean reads were mapped to the transcriptome reference database using TopHat and cufflinks software, allowing mismatches of no more than two bases. The unique mapped reads were used in subsequent analyses. The gene expression level was calculated using the FPKM method (fragments per kb per Million reads). Baggerley’s test and the false discovery rate (FDR) with a significance level of ≤0.001 and the absolute value of Log_2_Ratio ≥ 1 was set as the threshold to determine the significance of the gene expression difference. GO enrichment analysis was performed by first mapping all DEGs to GO terms in the database^3^, calculating the gene numbers for every term, and subsequently using the hypergeometric test to identify significantly enriched GO terms in the input list of DEGs, based on GO::TermFinder^4^.

### Weighted Gene Co-expression Network Analysis

To further analyze the DEGs, the strategy of weighted gene co-expression network (WGCNA) was used to identify the key genes. Co-expression focused on dynamic changes in the gene expression of samples at different times and calculates the topological overlap value. The WGCNA package in R language was used in the present study. Firstly, the FPKM values for all genes were sorted. Reflecting the excessive number of genes, the running of WGCNA package in R language was conducted under insufficient memory. According to the computer configuration, we removed the genes with low expression in the seven samples. Secondly, in the 13 groups of differences (e.g., P0 vs. CK3, P0 vs. T3, CK3 vs. T3, etc.), only genes showing differences of expression in more than five groups were further analysis. Finally, 8805 genes were analyzed using the WGCNA package. Fourteen modules were clustered. A heatmap of the expression of genes in each module was obtained using Mev 4.0. The correlation network by WCGNA analysis was showed in the Cytoscape.

### Validation and Expression Analysis of Quantitative Real-Time PCR

Total RNA used for Q-PCR analysis was extracted from the flowers/fruits at four different stages, i.e., 0, 3, 7, and 14 days, using three biological replicates of approximately 210 flowers/fruits. Total RNA was extracted as described above. First-strand cDNA was synthesized from 1 μg of total RNA using the PrimerScript^TM^ RT reagent Kit with gDNA Eraser (RR047, Takara, Japan), diluted 10 times with H_2_O and subsequently used as templates for Q-PCR assays. The Q-PCR mixture (15 μl total volume) contained 7.5 μl of SYBR Premix ExTaq^TM^ (RR820, Takara, Japan), 0.5 μl of each primer (10 μM), 2 μl of cDNA, and 4.5 μl of RNase-free water. The reactions were performed on a LightCycler480 instrument (Roche, Basel, Switzerland) according to the manufacturer’s instructions. The two-step Q-PCR program was initiated at 95°C for 30 s, followed by 40 cycles at 95°C for 5 s and 60°C for 20 s. Template-less controls for each primer pair were included in each run. The specificity of the Q-PCR primers was confirmed using a melting curve and the Q-PCR products were sequenced (Supplementary Table S1). Expression was calculated as 2^-ΔΔCt^ and normalized to that of the actin gene (JN004223) and 18 s rRNA (KY054923), and the data were managed using the Data Processing System (DPS, v. 7.05).

## Results

### Sequencing, *De novo* Assembly, and Gene Annotation in RNA-Seq of Loquat Fruits

**FIGURE 1:**
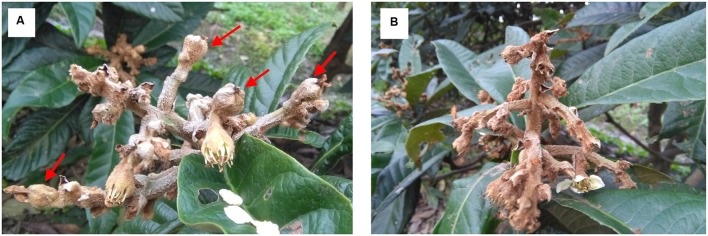
**Development of the triploid loquat at 3 weeks after gibberellin (GA) treatment.** **(A)** GA treatment. **(B)** Control.

**FIGURE 2:**
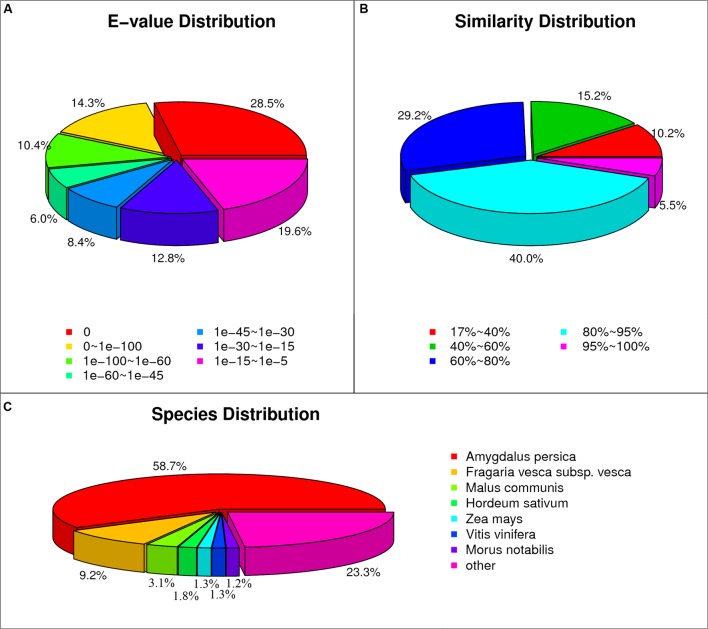
**Characteristics of the homology search of loquat unigenes against the non-redundant (nr) database.** **(A)** *E*-value distribution of the top BLAST hits for each unique sequence. **(B)** Similarity distribution of the top BLAST hits for each unique sequence. **(C)** Species distribution of the top BLAST hits for all homologous sequences.

The flowers/fruits of triploid loquat were browning, losing water and beginning to drop at 2 weeks after peak flowering under normal conditions. In the present study, more than four fruits in one inflorescence with GA treatment showed the closure of the calyx at 3 weeks, while all fruits of control were gradually dropped (**Figure 1**). Seven samples (P0, CK3, CK7, CK14, T3, T7, and T14) were subjected to total RNA extraction and RNA-Seq analysis. High-throughput sequencing generated 60.24–64.29 million (M) pairs of 150 bp raw reads from each library. After a stringent quality filtering process, 344 million clean reads (79.52% of the raw data) remained, with a Q30 percentage (an error probability lower than 0.1%) ranging from 92.62 to 93.17%. The number of clean reads per library ranged from 47.62 to 50.83 M (**Table 1**). The total clean reads were *de novo* assembled into transcripts by trinity, and 153,900 genes were assembled with an average length of 1,188 bp (N50 = 1942). All unique sequences were annotated based on BLASTx (cut-off *E*-value 10^-5^) searches of four public databases: NCBI non-redundant (nr) database, Swiss-Prot protein database, KEGG database, and GO database. Among these unique sequences, 122,966 known genes (79.9% of the total genes) and 30,934 new transcripts were identified. Based on the nr annotations, 53.2% of the annotated sequences had strong homology (*E*-value < 10^-60^), 14.4% of the annotated sequences showed strong homology (10^-60^ < *E*-value < 10^-30^), and an additional 32.4% of the annotated sequences showed homology (10^-30^ < *E*-value < 10^-5^) to available plant sequences (**Figure 2A**). The similarity distribution was comparable, with 45.5% of the sequences having similarities higher than 80%, while 54.6% of the hits had similarities of 17–80% (**Figure 2B**). With respect to species, 58.7% of the unique sequences had top matches to sequences from *Amygdalus persica*, with additional hits to *Fragaria vesca subsp. vesca* (9.2%), *Malus communis* (3.1%), and *Hordeum sativum* (1.8%) (**Figure 2C**). We used COG, GO, and KEGG assignments to classify the functions of the predicted pear unigenes. Approximately 52,581 sequences could be annotated in COG function classification (**Supplementary Figure S1**). A total of 41,879 sequences could be annotated using GO and were categorized into three main categories: biological processes, cellular components, and molecular functions. A total of 71,180 unigenes (∼46.3%) mapped to 128 KEGG pathways (Supplementary Table S2). The maps with highest unigene representation were metabolic pathways (KO01100; 17,711 unigenes, 24.9%), followed by biosynthesis of secondary metabolites (KO01110; 7,476 unigenes, 10.5%), plant-pathogen interactions (KO04626; 4,305 unigenes, 6.1%), and RNA transport (KO03013; 3,705 unigenes, 5.2%).

**Table 1:** Statistics of the reads in the present study.

Samples	Raw reads	Total clean reads	Q20	Q30
P0	64,287,816	50,476,668	97.16%	93.17%
CK3	62,631,268	50,834,572	96.94%	92.72%
CK7	60,736,874	48,326,940	96.99%	92.81%
CK14	63,073,246	49,527,576	96.99%	92.81%
T3	60,410,780	47,623,684	97.06%	92.94%
T7	60,243,284	48,326,940	96.90%	92.62%
T14	62,000,208	49,527,576	97.00%	92.82%


### Identification of DEGs in GA Treatment

**FIGURE 3:**
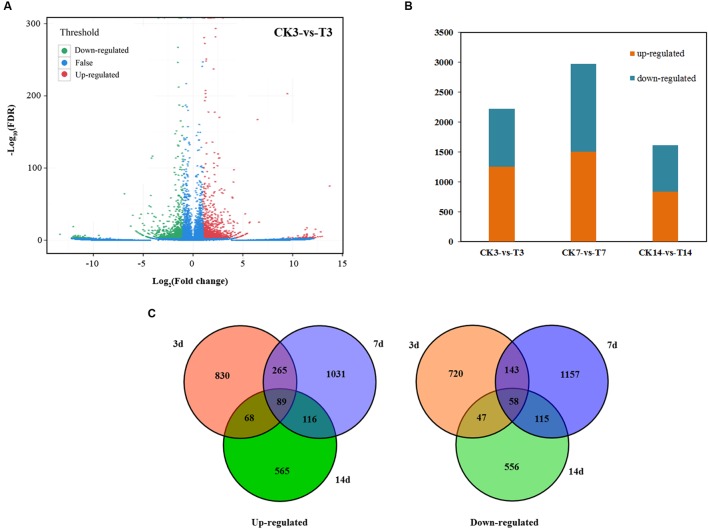
**Statistics of differently expressed genes.** **(A)** Significantly up- or downregulated genes using the threshold of FDR ≤ 0.001 and log_2_Ratio ≥ 1 in CK3 vs. T3. **(B)** Graphical representation of overall differently expressed genes in GA treatment. **(C)** Number of upregulated and downregulated transcripts in GA treatment illustrated using a Venn diagram.

Seven DGE libraries were sequenced, and the FPKM (fragments per kb per million fragments) of all unigenes were calculated at four stages in loquat fruit after GA treatment. Differences in gene expression in seven samples were examined using the threshold of FDR ≤ 0.001 and |log_2_Ratio|≥ 1. The DEGs were identified by pairwise comparisons of the seven libraries, i.e., P0 vs. CK, CK3 vs. T3, CK7 vs. T7, and CK14 vs. T14 (**Figure 3A**). A total of 5,760 DEGs were detected between the CK and T libraries, with 2,964 upregulated and 2,796 downregulated genes (**Figure 3B**). For each stage, 2,220 DEGs were detected between the CK3 and T3 libraries. These genes were directly affected by GA treatment. In CK7 vs. T7, 2,974 DEGs were identified, which is more than in CK3 vs. T3 and CK14 vs. T14 (1,614 DEGs; **Figure 3B**). Several DEGs showed a trend of increasing and decreasing. Interestingly, 89 transcripts were commonly upregulated at all time points, as illustrated in the Venn diagram (**Figure 3C**; Supplementary Table S2), while 58 common transcripts were downregulated (**Figure 3C**; Supplementary Table S2). We used GO assignments to classify the functions of DEGs in pairwise comparisons. Most genes in reproduction, reproductive process, extracellular region, and macromolecular complex were upregulated, but genes in immune system process and nutrient reservoir activity were mostly downregulated (**Supplementary Figure S2**). The analysis of the top 20 KEGG pathways showed that the metabolic pathway was the main biochemical metabolism, with a large number of identified genes (**Supplementary Figure S3**). The plant hormone signal transduction pathway was also active. In contrast with the samples at 3 and 7 days after GA treatment, the zeatin biosynthesis and starch and sucrose metabolism pathways were significantly enriched in 14 days.

### Weighted Gene Co-expression Network Analysis (WCGNA) of DEGs

The WCGNA analysis was used to identify the expressed module of genes. Using this method, we identified some interesting modules related to GA treatment, which was efficient to identify the key genes. A total of 8805 DEGs were clustered in 14 modules. We printed a heatmap of gene expression in each module (**Supplementary Figure S4**) and a correlation network by WCGNA analysis in the Cytoscape (**Supplementary Figure S5**). A large number of unigenes belongs to the Blue (1,660 DEGs), Brown (1,405 DEGs), and Turquoise (2,685 DEGs) modules, but the trend of gene expression was not relative to the treatment in the present study. In the modules in Tan, 64 unigenes were isolated (Supplementary Table S3), and the expression module of these genes in treatment and control was opposite (**Supplementary Figure S4**). We annotated all 64 genes in the module in Tan using NR, NT, and Swiss-Prot databases (Supplementary Table S3).

### Putative Related Genes in Loquat Fruit Setting and Development with GA Treatment

**FIGURE 4:**
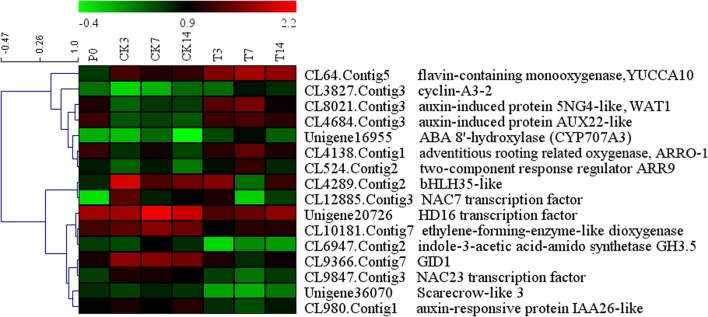
**Heatmap of the expression of genes related to fruit setting**.

**FIGURE 5:**
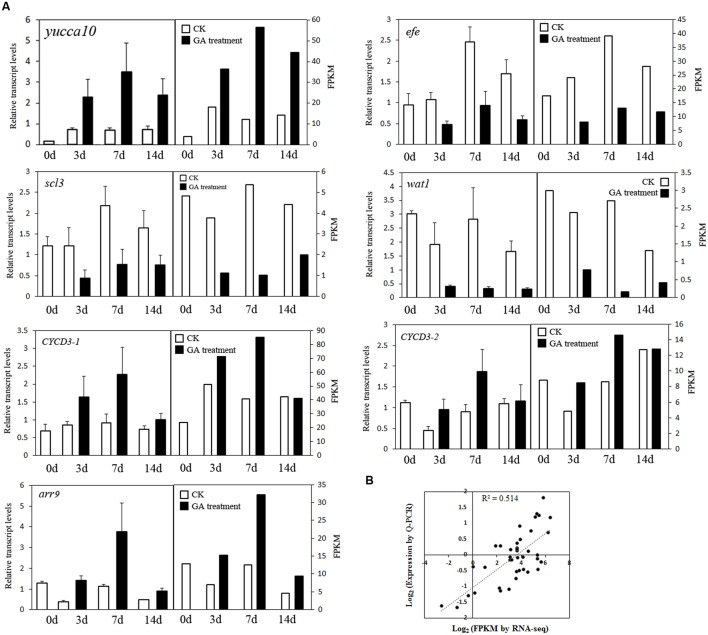
**The expression of seven genes after GA treatment.** **(A)** Q-PCR validation of differential gene expression. **(B)** Coefficient analysis between the gene expression ratios obtained from RNA-seq and Q-PCR data.

We combined the DEGs identified from the Venn diagram and WCGNA (Supplementary Tables S2 and S3), and most DEGs identified from these two methods overlapped. All DEGs were annotated and further analyzed. Sixteen genes (seven upregulated genes and nine downregulated genes) were identified and may play important roles in fruit setting (**Figure 4**). Five genes were related to auxin. One gene (CL64, *yucca10*) synthesizes auxin, and four genes (CL8021, *wat1*; CL4684, *aux22*; CL980, *IAA26*, and CL6947, *GH3.5*) are induced/responded by auxin, in which two genes (*wat1* and *AUX22*) were upregulated, and the other two genes (*IAA26* and *GH3.5*) were downregulated. One gene of ABA 8′-hydroxylase (Unigene16955, *CYP707A3*) related to oxidative catabolism of ABA and one gene of adventitious rooting related oxygenase (CL4138, *ARRO-1*) were upregulated. Two genes (CL3827, *CYCA3* and CL524, *arr9*) were related to cell division, and upregulated after GA treatment. In downregulated genes, one gene, *efe* (ethylene-forming enzyme dioxygenase), regulates ethylene synthesis. The gibberellin signaling gene SCARECROW-LIKE 3 (Unigene36070, *scl3*) and the GA receptor gene GIBBERELLIN INSENSITIVE DWARF1 (CL9366, *gid1*) were identified. Four transcription factors were downregulated: *bHLH35*, *NAC7*, *NAC23*, and *HD16*. In addition, three genes (CL5408, CL9101, and Unigene40276) were upregulated and related to the ubiquitin degradation pathway (Supplementary Table S3). Eleven genes (geneID ranged from 9 to 19 in Supplementary Table S3) were annotated to proteins involved in the formation of the cell wall, such as xyloglucan endotransglucosylase (CL10207), expansin (CL15890), and leucine-rich repeat extension (CL2524), and most of these genes were upregulated. The expression of seven genes was confirmed using Q-PCR (**Figure 5A**), indicating good reproducibility with RNA-seq data (**Figure 5B**).

GIBBERELLIN INSENSITIVE DWARF1 and DELLA proteins were reported to regulate GA signaling. In the present study, we analyzed the expression of all *gid1* and *della* genes. According to the annotation of KEGG, eight *gid1* (KEGG: K14493) and 18 *della* genes (KEGG: K14494) were isolated. The heatmap of the expression of these genes showed that three members in *della* (CL10545, CL5461, and CL8453) were highly expressed (**Supplementary Figure S6**). The expression of *della* was stable in the control and treatment samples. Two members of *gid1* (CL9366 and CL11542) were annotated, and the expression of these genes was upregulated in the control and downregulated after GA treatment.

## Discussion

In plant development, pollination and fertilization are the first steps in the shift of flowers to fruits. The egg cell in the ovary is fertilized by pollen to induce seeds. The zygote potentially triggers the development of the ovary into a fruit. Fruit setting is therefore dependent on certain growth signals generated by fertilization. Triploid loquat is a hybrid with 17 chromosomes from the male parent, and 34 from the maternal parent (Wang et al., 2011a). It has a low fruit setting rates in the nature because it has no seed. Thus, triploid loquat is an outstanding resource with a high edible rate. The application of GAs could induce parthenocarpy in triploid loquat and promote seedless fruit. In the present study, a triploid loquat cDNA library and seven DGE libraries (from samples collected at 0, 3, 7, and 14 days after GA treatment) were constructed using RNA-Seq technology and used to screen DEGs during fruit setting after GA treatment. We obtained 153,900 unique sequences, of which 122,966 sequences could be annotated, including 76,586 clusters and 77,314 singletons. In GenBank, only 2242 nucleotide sequences of *Eriobotrya* plants were deposited until July 2016. The results of the present study would be helpful to clone gene sequences and analyze gene families in loquat. To our knowledge, this study is the first report to use RNA-Seq techniques to identify large numbers of genes involved in different metabolic pathways in loquat fruit setting.

A large number of DEGs were differentially expressed during fruit setting after GA treatment through RNA-seq analysis. A total of 2,220, 2,974, and 1,614 genes were differentially expressed at 3, 7, and 14 days after GA treatment, respectively. These results showed that the number of DEGs increased from 3 to 7 days and subsequently decreased at 14 days. By analyzing KEGG pathways, we identified DEGs that participated in several different pathways. In contrast with the samples in examined at 3 and 7 days after treatment, the pathways of zeatin biosynthesis and starch and sucrose metabolism were significantly enriched at 14 days, suggesting that these pathways may be regulated by a GA signal in the development of loquat fruit at 2 weeks.

**FIGURE 6:**
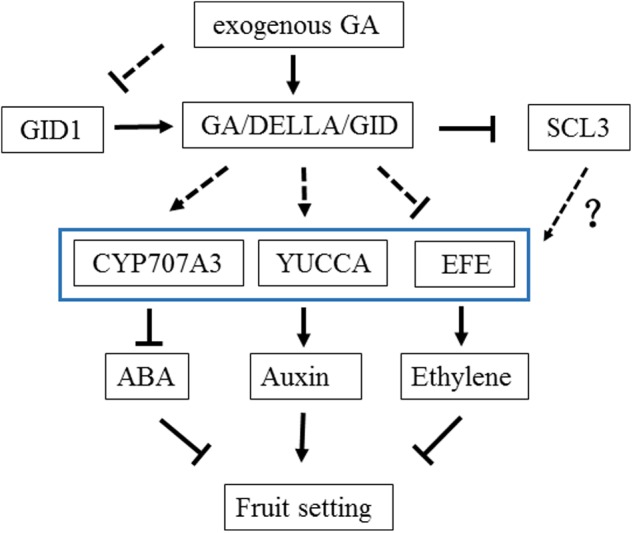
**A model for fruit setting in the treatment of GA in loquat**.

In the present study, two methods (Venn diagram and WCGNA analysis) were used to screen DEGs to identify critical genes in GA treatment. Two methods were better to identify the DEGs. Sixteen genes were putatively related to GA treatment (**Figure 4**). Three genes may play important roles in the fruit setting of triploid loquat. Firstly, an ethylene-forming enzyme [EFE, sometimes named as 1-aminocyclopropane-1-carboxylic acid oxidase (ACO)] was downregulated after GA treatment. EFE catalyzes the last step in the biosynthesis of the plant hormone ethylene (Hamilton et al., 1991). The ethylene effects include the forced ripening of fruits (Picton et al., 1993), and the ethephon (ethylene releasing compound) is used for fruit thinning in many fruit trees (Jones et al., 1990; Wertheim, 2000). The high expression of *efe* may induce ethylene biosynthesis and subsequently induce fruit dropping. Secondly, the flavin-containing monooxygenase *yucca*10 was upregulated after GA treatment. The roles of YUCCA genes in local auxin biosynthesis and plant development have been reported (Cheng et al., 2006). YUCCA regulates the initiation of floral meristems and lateral organs during vegetative and reproductive development (Gallavotti et al., 2008). The tissues of unfertilized and depistillated flowers significantly accumulated with lower levels of auxin (Brcko et al., 2012). Several studies have reported an increase in the auxin concentration in the ovary after GA treatment (Sastry and Muir, 1963; Niu et al., 2014). GAs may play a role in increasing auxin production in the ovary, which in turn may act as a signal for fruit setting. Thirdly, the CYP707A3 gene (ABA 8′-hydroxylase) was upregulated. This gene has been implicated as responsible for the rapid decrease in ABA level (Kushiro et al., 2004). The high level of ABA promotes fruit dropping. With GA treatment, the upregulation of *CYP707A3* is a benefit to fruit setting. These results confirmed that auxin, ethylene, and abscisic acid are involved in regulating fruit setting, consistent with tomato (Vriezen et al., 2008). Under natural conditions, the expression of *efe* was high in the fruit of triploid loquat. The fruit dropped at 2 weeks after flowering, consistent with a potential burst of ethylene. When the fruits were sprayed with GA, the expression of *efe* was suppressed. In addition, the *yucca* and *CYP707A3* were stimulated (**Figure 6**), suggesting that these three hormones may be involved to fruit setting in GA treatment.

Other genes identified in the present study have been previously identified in other perennial plants. In *Arabidopsis*, the expression of A-type cyclins (*CYCA3;1* and *CYCA3;2*) was observed in actively dividing tissues, such as root and shoot apical meristems and lateral root primordia (Takahashi et al., 2010). In our study, *CYCA3;2* was upregulated, suggesting that the meristem of loquat fruit was divided. Another cyclin gene, *Cyclin D3*, was also upregulated under GA treatment, which induces mitotic cell division and reduces endoreduplication (Schnittger et al., 2002; Dewitte et al., 2003). One type-A gene of the *Arabidopsis* response regulators (*arr9*) was upregulated, which are rapidly induced by cytokinin and are highly similar to bacterial two-component response regulators (To et al., 2004). It has been suggested that cytokinins might be involved in the regulation of the fruit setting in GA treatment. Four auxin-related genes were isolated. Upregulated *AUX22* and downregulated *IAA26* were the members of the Aux/IAA gene family, suggesting that this family has a different regulation pattern in the auxin response. *wat1* was induced by auxin and promoted cell wall thickness (Ranocha et al., 2011), which was upregulated after GA treatment. *GH3.5* catalyzes the synthesis of indole-3-acetic acid (IAA)-amino acid conjugates, providing a mechanism for the plant to cope with the presence of excess auxin. The downregulation of *GH3.5* may have induced high levels of IAA. Auxin-responsive genes may play an important role in fruit setting with GA treatment. Generally, adventitious rooting related oxygenase (*ARRO-1*) induces adventitious root formation in apple stem disks (Butler and Gallagher, 1999). In the present study, one gene of *ARRO-1* was upregulated in fruit setting, suggesting that the involvement of this gene in tissue division is not only significant in root formation but also in fruit setting. In GA signaling downstream, a large number of genes related to the cell growth were upregulated (Supplementary Table S3), promoting cell division and inducing parthenocarpy.

GIBBERELLIN INSENSITIVE DWARF1 and DELLA are associated with GA signaling in plants (Ueguchitanaka et al., 2007). Recent studies have reported that the triple complex of GID1-GA-DELLA is in turn targeted by the SCF^GID2^ complex, and the DELLA protein is degraded, which releases the repressive state of GA responses (Ueguchi-Tanaka et al., 2005). In the present study, all members of *gid1* in the transcriptome were isolated, and the expression of these genes was suppressed with GA treatment. A negative feedback mechanism in GA signaling may be regulated by *gid1*. When exogenous GAs were administered, *gid1* was suppressed to inhibit the combination of GID1-GA-DELLA, thus reducing the impact of GAs. All *della* genes were isolated from the transcriptome, and the expression levels were also investigated (**Supplementary Figure S6**). The value of FPKM in different members was stable, suggesting that the *della* may not be involved in feedback regulation under GA treatment. The downstream of GA signaling gene of *scl3* was downregulated. Scarecrow-like 3 promotes gibberellin signaling by antagonizing the master growth repressor DELLA in *Arabidopsis* (Zhang et al., 2011), and it controls *Arabidopsis thaliana* meristem size (Moubayidin et al., 2016). The DELLA signaling pathway for the suppression of ethylene synthesis and promotion of auxin synthesis remains unclear. *scl3* may be involved in this regulation, which should be further analyzed.

In addition, differentially regulated transcription factors, including *NAC7*, *NAC23*, *bHLH35*, and *HD16*, were identified in the present study. Among these factors, *NAC* has previously been reported in formation of apical meristem and hormone regulation (Duval et al., 2002; Kim et al., 2008). The present study showed that two members of *NAC* were negatively regulated by GA in the fruit setting of loquat. In *Populus*, *bHLH35* was induced by drought and abscisic acid (Dong et al., 2014). With GA treatment in loquat, the suppression of *bHLH35* might be related with the upregulation of *CYP707A3*. Based on DGE analysis, the expression levels of the genes encoding these transcription factors significantly changed during GA treatment in loquat.

## Conclusion

To our knowledge, this work is the first study to provide comprehensive sequencing and DGE profiling data for a dynamic view of transcriptomic variation in loquat fruit setting after treatment with exogenous GA_3_. Approximately 5760 genes involved in many metabolic processes were significant differentially regulated 2 weeks after GA treatment. Genes related to fruit setting and their expression profiles at four stages were analyzed further, offering new insights into the molecular mechanisms underlying loquat fruit setting. Sixteen candidate genes were identified that may play important roles in GA signaling, including hormone-related genes, transcription factors, and cell division genes. Our findings provided a relatively complete molecular platform for future studies on the progression of fruit setting.

## Availability of Data and Materials

The SRA data of RNA-seq was deposited in NCBI, and the rest of data supporting our findings is contained within the manuscript and supplementary files.

## Author Contributions

SJ and FX performed the experiments and wrote the manuscript. JL helped to revise the manuscript. XZ involved in designing the research and revised the manuscript. All authors read and approved the manuscript.

## Conflict of Interest Statement

The authors declare that the research was conducted in the absence of any commercial or financial relationships that could be construed as a potential conflict of interest.

## Footnotes

1 http://www.ncbi.nlm.nih.gov/sra/

2 http://pan.baidu.com/s/1dEROH81, password: sn9m

3 http://www.geneontology.org/

4 http://search.cpan.org/∼sherlock/GO-TermFinder/

## Supplementary Material

**FIGURE S1 | COG function classification of all unigenes.**

**FIGURE S2 | Histogram of gene ontology classifications of DEGs in CK3 vs. T3, CK7 vs. T7, and CK14 vs. T14.**

**FIGURE S3 | Kyoto Encyclopedia of Genes and Genomes pathways in CK3 vs. T3, CK7 vs. T7, and CK14 vs. T14.**

**FIGURE S4 | Heatmap of the expression of genes in the 14 modules.**

**FIGURE S5 | Correlation network by WCGNA analysis in the Cytoscape based on 8805 DEGs.**

**FIGURE S6 | Heatmap of the expression of members in *gid1* and *della*.**
