# Supplementary figures and images for: Transcriptome Analysis Reveals Candidate Genes Involved in Gibberellin-Induced Fruit Setting in Triploid Loquat (Eriobotrya japonica)
Source: Front Plant Sci. 2016 Dec 21;7:1924. doi: 10.3389/fpls.2016.01924 (PMC5174095; doi:10.3389/fpls.2016.01924)

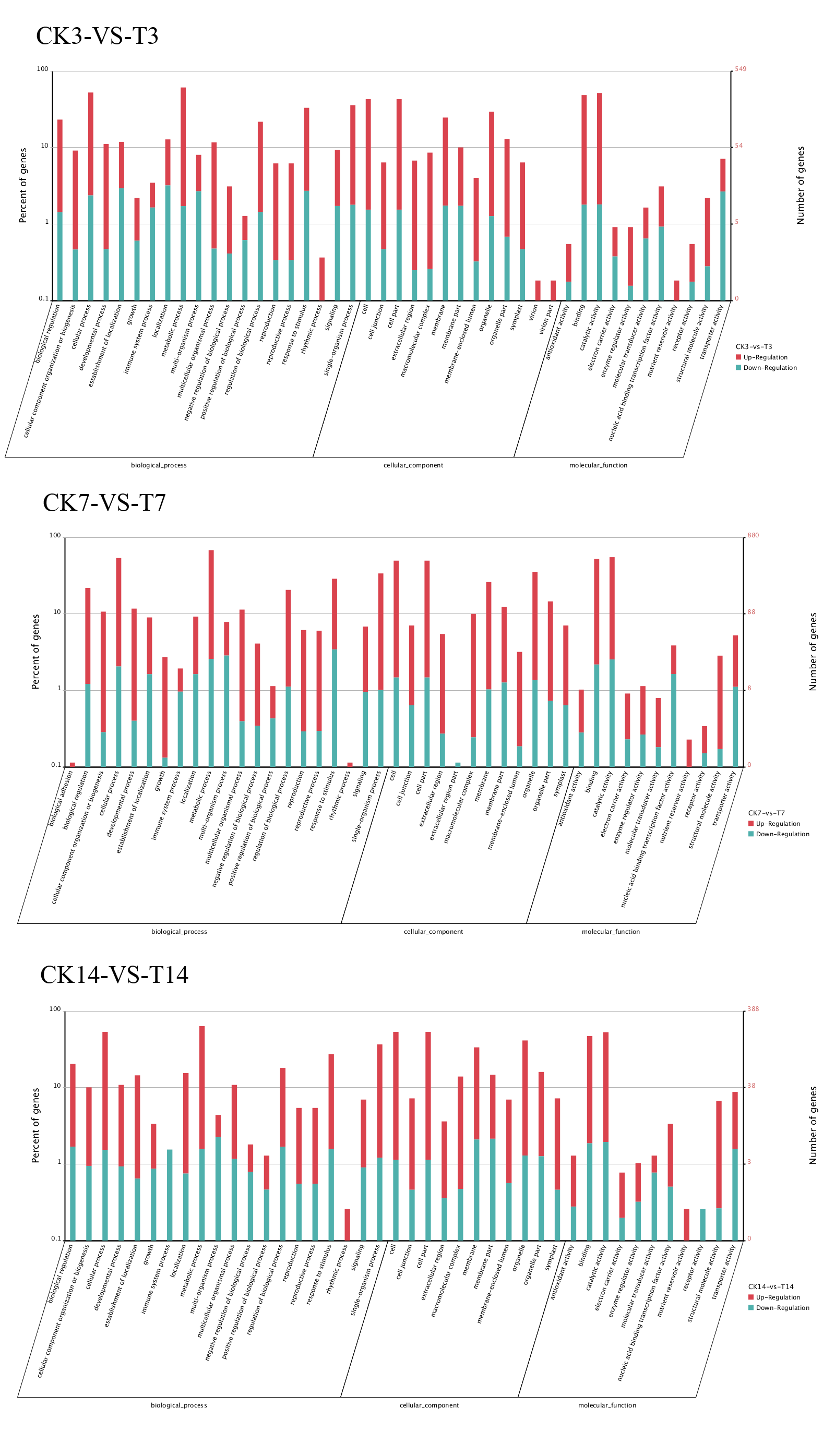

Supplement: Supplementary file 2 [file Image_2.TIF]

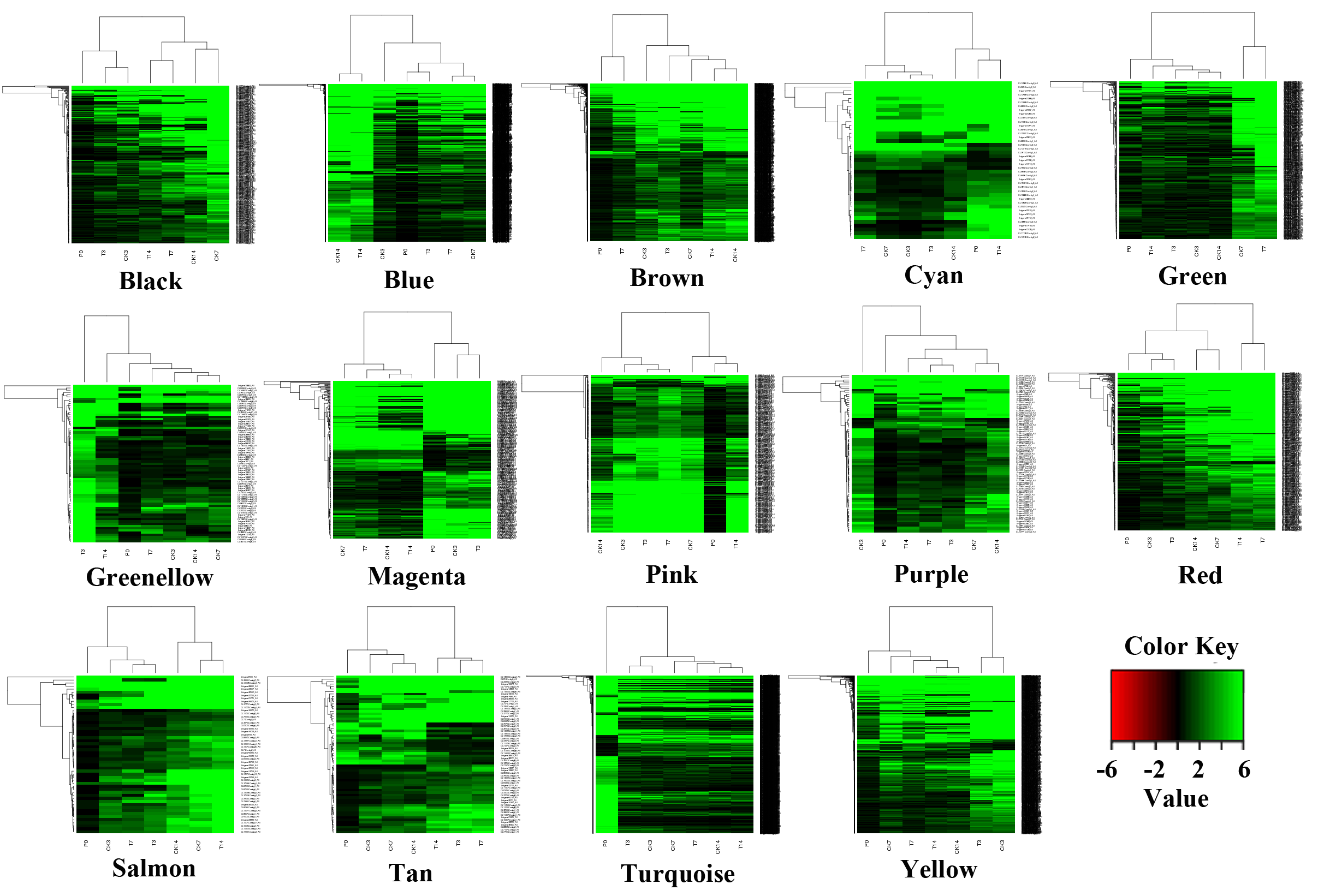

Supplement: Supplementary file 4 [file Image_4.TIF]

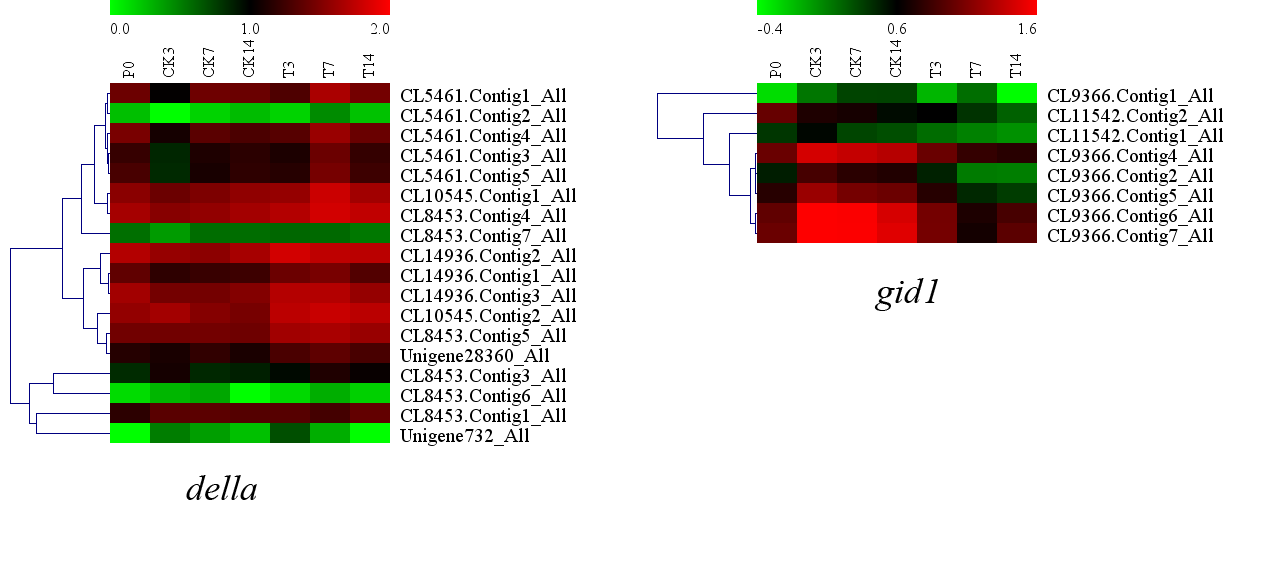

Supplement: Supplementary file 6 [file Image_6.TIF]
