# Supplementary material for: Transcriptome Analysis Reveals Candidate Genes Involved in Gibberellin-Induced Fruit Setting in Triploid Loquat (Eriobotrya japonica)
Source: Front Plant Sci. 2016 Dec 21;7:1924. doi: 10.3389/fpls.2016.01924 (PMC5174095; doi:10.3389/fpls.2016.01924)
Supplement: Supplementary file 3 [file Image_3.pdf]

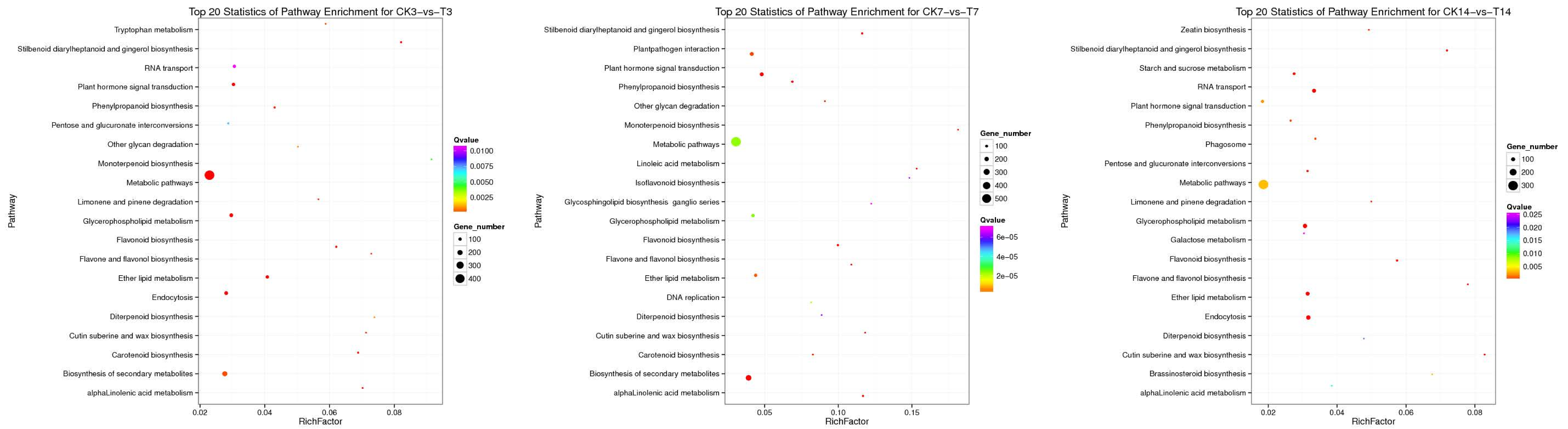
Top 20 Statistics of Pathway Enrichment for CK3–vs–T3
Tryptophan metabolism
Stilbenoid diarylheptanoid and gingerol biosynthesis
RNA transport
Plant hormone signal transduction
Phenylpropanoid biosynthesis
Pentose and glucuronate interconversions
Other glycan degradation
Monoterpenoid biosynthesis
Metabolic pathways
Limonene and pinene degradation
Glycerophospholipid metabolism
Flavonoid biosynthesis
Flavone and flavonol biosynthesis
Ether lipid metabolism
Endocytosis
Diterpenoid biosynthesis
Cutin suberine and wax biosynthesis
Carotenoid biosynthesis
Biosynthesis of secondary metabolites
alphaLinolenic acid metabolism
Pathway
RichFactor
0.02
0.04
0.06
0.08
Qvalue
0.0100
0.0075
0.0050
0.0025
Gene_number
100
200
300
400
Top 20 Statistics of Pathway Enrichment for CK7–vs–T7
Stilbenoid diarylheptanoid and gingerol biosynthesis
Plantpathogen interaction
Plant hormone signal transduction
Phenylpropanoid biosynthesis
Other glycan degradation
Monoterpenoid biosynthesis
Metabolic pathways
Linoleic acid metabolism
Isoflavonoid biosynthesis
Glycosphingolipid biosynthesis ganglio series
Glycerophospholipid metabolism
Flavonoid biosynthesis
Flavone and flavonol biosynthesis
Ether lipid metabolism
DNA replication
Diterpenoid biosynthesis
Cutin suberine and wax biosynthesis
Carotenoid biosynthesis
Biosynthesis of secondary metabolites
alphaLinolenic acid metabolism
Pathway
RichFactor
0.05
0.10
0.15
Gene_number
100
200
300
400
500
Qvalue
6e–05
4e–05
2e–05
Top 20 Statistics of Pathway Enrichment for CK14–vs–T14
Zeatin biosynthesis
Stilbenoid diarylheptanoid and gingerol biosynthesis
Starch and sucrose metabolism
RNA transport
Plant hormone signal transduction
Phenylpropanoid biosynthesis
Phagosome
Pentose and glucuronate interconversions
Metabolic pathways
Limonene and pinene degradation
Glycerophospholipid metabolism
Galactose metabolism
Flavonoid biosynthesis
Flavone and flavonol biosynthesis
Ether lipid metabolism
Endocytosis
Diterpenoid biosynthesis
Cutin suberine and wax biosynthesis
Brassinosteroid biosynthesis
alphaLinolenic acid metabolism
Pathway
RichFactor
0.02
0.04
0.06
0.08
Gene_number
100
200
300
Qvalue
0.025
0.020
0.015
0.010
0.005
